# LncRNA NEAT1 Potentiates SREBP2 Activity to Promote Inflammatory Macrophage Activation and Limit Hantaan Virus Propagation

**DOI:** 10.3389/fmicb.2022.849020

**Published:** 2022-04-13

**Authors:** Yongheng Yang, Mengyun Li, Yongtao Ma, Wei Ye, Yue Si, Xuyang Zheng, He Liu, Linfeng Cheng, Liang Zhang, Hui Zhang, Xijing Zhang, Yingfeng Lei, Lixin Shen, Fanglin Zhang, Hongwei Ma

**Affiliations:** ^1^College of Life Sciences, Northwest University, Xi'an, China; ^2^Department of Microbiology, School of Basic Medicine, The Fourth Military Medical University, Xi'an, China; ^3^Department of Emergency, Children's Hospital of Kaifeng City, Kaifeng, China; ^4^Department of Infectious Diseases, Tangdu Hospital, The Fourth Military Medical University, Xi'an, China; ^5^Department of Anesthesiology and Critical Care Medicine, Xijing Hospital, The Fourth Military Medical University, Xi'an, China

**Keywords:** hantavirus, Hantaan virus, lncRNA, NEAT1, SREBP2, inflammatory macrophage, HFRS

## Abstract

As the global prototypical zoonotic hantavirus, Hantaan virus (HTNV) is prevalent in Asia and is the leading causative agent of severe hemorrhagic fever with renal syndrome (HFRS), which has profound morbidity and mortality. Macrophages are crucial components of the host innate immune system and serve as the first line of defense against HTNV infection. Previous studies indicated that the viral replication efficiency in macrophages determines hantavirus pathogenicity, but it remains unknown which factor manipulates the macrophage activation pattern and the virus-host interaction process. Here, we performed the transcriptomic analysis of HTNV-infected mouse bone marrow-derived macrophages and identified the long noncoding RNA (lncRNA) nuclear enriched abundant transcript 1 (NEAT1), especially the isoform NEAT1-2, as one of the lncRNAs that is differentially expressed at the early phase. Based on coculture experiments, we revealed that silencing NEAT1-2 hinders inflammatory macrophage activation and facilitates HTNV propagation, while enhancing NEAT1-2 transcription effectively restrains viral replication. Furthermore, sterol response element binding factor-2 (SREBP2), which controls the cholesterol metabolism process, was found to stimulate macrophages by promoting the production of multiple inflammatory cytokines upon HTNV infection. NEAT1-2 could potentiate SREBP2 activity by upregulating *Srebf1* expression and interacting with SREBP2, thus stimulating inflammatory macrophages and limiting HTNV propagation. More importantly, we demonstrated that the NEAT1-2 expression level in patient monocytes was negatively correlated with viral load and HFRS disease progression. Our results identified a function and mechanism of action for the lncRNA NEAT1 in heightening SREBP2-mediated macrophage activation to restrain hantaviral propagation and revealed the association of NEAT1 with HFRS severity.

## Introduction

Hantaviruses (order *Bunyavirales*, family *Hantaviridae*) are monopartite, trisegmented, negative-stranded enveloped RNA viruses (-ssRNA). The hantaviral genome is composed of three segments, named small (S), medium (M), and large (L), which encode the nucleoprotein (NP), glycoprotein precursor (GPC, cleaved into the N-terminal Gn/G1 and C-terminal Gc/G2) and viral RNA-dependent RNA polymerase (RdRp), respectively (Jonsson et al., 2010). As rodent-borne pathogens, hantaviruses cause two types of viral hemorrhagic fevers in humans, namely, hemorrhagic fever with renal syndrome (HFRS), which is prevalent in Eurasia, and hantavirus pulmonary syndrome (HPS), which is prevalent in the Americas; the fatality rates of HFRS and HPS reach 0.1%–15% and 20%–40%, respectively (Saavedra et al., 2021). Increased numbers of hantavirus disease cases have recently considerably threatened global public health and have drawn worldwide attention (Li et al., 2021). Approximately 20,000 cases are registered annually worldwide, and the majority of these are HFRS caused by the Old World hantaviruses (such as Hantaan virus/HTNV and Puumala virus/PUUV; Jiang et al., 2017). According to the monitoring data of the Chinese Center for Disease Control and Prevention (CDCD), in the last decade (2010–2020), the annual incidence rate of HFRS in China ranged from 0.58 to 1.24 cases/100,000 persons (Chinese National Bureau of Statistics, 2020), contributing to more than 80% of total global casualties. HTNV, the prototypical hantavirus, is the etiological agent of severe HFRS. To date, neither effective therapeutics nor preventive strategies against HTNV infection have been developed (Liu et al., 2019). Hence, it is of great importance to elucidate the decisive determinants of HTNV-associated pathogenicity.

Mononuclear phagocytes (MNPs), which include monocytes and macrophages, were shown to facilitate hantaviral propagation and the immunopathogenesis of HFRS both *in vivo* and *in vitro* (Scholz et al., 2017; Raftery et al., 2020; Vangeti et al., 2021). The replicative efficiency of hantaviruses in MNPs determines their pathogenicity in humans (Raftery et al., 2020), and MNPs were also found to carry and spread PUUV from patient blood to the airway and kidneys during the acute phase of hantavirus disease (Scholz et al., 2017; Vangeti et al., 2021). Exploring the endogenous anti-hantaviral factors in MNPs might reveal novel therapeutic avenues for HFRS. The classical inflammatory activation pattern of macrophages (M1 polarization) or monocytes (M1-like monocytes), which is stimulated by host Th1-type cytokines (such as interferon-γ/IFNγ and tumor necrosis factor-α/TNFα) or pathogen-associated molecular patterns (PAMPs) *via* JAK/STAT, NF-κB or pattern-recognition receptor (PRR)-mediated signaling, maintains the potential ability of the immune system to restrict viral infection and promote virus clearance by initiating a robust interferon-centered inflammation response, the dysregulation of which triggers a cytokine storm and aggravates host immunopathological injury (McNab et al., 2015; Merad and Martin, 2020; McGonagle et al., 2021). Alternatively, macrophages can be M2-polarized by Th2-type cytokines (such as IL-4 and IL-10), which promote the resolution of inflammatory responses and assist in tissue repair (Wang et al., 2021). It has been reported that hantavirus (strain no. 60343) can enhance bactericidal activity in macrophages by upregulating oxygen-dependent metabolism and NO synthase (Plekhova et al., 2005). Our previous study also showed that HTNV induced the formation of an inflammasome complex centered on the nucleotide-binding domain leucine-rich repeat-containing protein 3 (NLRP3), thus promoting the secretion of interleukin-1β (IL-1β) and the release of reactive oxygen species (ROS) in monocytes (Ye et al., 2015). It remains unclear whether metabolic process could affect the activation status of macrophages or monocytes upon hantaviral infection.

Sterol regulatory element-binding proteins (SREBPs, also known as SREB factors/SREBFs, encoded by *Srebf* genes) are pivotal transcription factors that respond to low sterol concentrations and regulate cellular lipid metabolism (Liu F. et al., 2021; Xu Y. et al., 2021). SREBPs have also been reported to be hijacked for replication by multiple viruses (Martín-Acebes et al., 2019; Cloherty et al., 2020; Tanner and Alfieri, 2021). SREBP1, encoded by *Srebf1* on human chromosome 17p11.2, preferentially regulates fatty acid levels, while SREBP2, encoded by *Srebf2* on 22q13, promotes cellular cholesterol synthesis by directly encoding a series of enzymes (Horton et al., 2002). Under cholesterol-depleted conditions, SREBP cleavage-activating proteins (SCAPs) are stimulated to escort SREBP2 from the endoplasmic reticulum (ER) membrane to the Golgi, where SREBP2 undergoes proteolytic cleavage in multiple steps to release the ∼480 amino acid NH_2_-terminal domain (N-SREBP2). N-SREBP2 is a mature, biologically active peptide that translocates into the nucleus, binds to sterol regulatory elements (SREs), and activates its canonical target genes (such as *7-dehydrocholesterol reductase*/*Dhcr7* and *squalene epoxidase/Sqle*) to promote cholesterol biosynthesis (Liu F. et al., 2021; Xu Y. et al., 2021). Recent studies revealed that SREB2 could not only promote NLRP3 inflammasome activation but also directly bind to inflammatory genes and trigger their expression, ultimately stimulating M1-type macrophages (Guo et al., 2018; Kusnadi et al., 2019). Little is known about how SREBP2-mediated macrophage activation is regulated.

The long noncoding RNA (lncRNA) nuclear enriched abundant transcript 1 (NEAT1), also known as nuclear paraspeckle assembly transcript 1, is transcribed from the multiple endocrine neoplasia locus (also known as MEN), regulates host inflammatory responses to stress, and is highly involved in tumor metastasis (Imamura et al., 2014; Dong et al., 2021; Ghafouri-Fard et al., 2021; Yamamoto et al., 2021). The NEAT1 gene is transcribed into two overlapping isoforms, namely, 3.7 kb NEAT1-1 (*MEN epsilon*) and 23.0 kb NEAT1-2 (*MEN beta*), which act as an essential scaffold for paraspeckles in the nucleus by assembling a group of RNA-binding proteins, such as paraspeckle component 1 (PSPC1), non-POU domain containing octamer binding (NONO), and splicing factor proline/glutamine-rich (SFPQ). Recent research showed that NEAT1-1 was transferred from the nucleus to the cytoplasm by Pinin after glucose stimulation, where it could recruit the PGK1/PGAM1/ENO1 complex for efficient glycolysis, promoting breast cancer tumorigenesis and metastasis (Park et al., 2021). NEAT1-2 expression was elevated through TLR3 or MDA5 pathway upon infection with several viruses, such as influenza virus or herpes simplex virus 1 (HSV-1), but not measles virus (MV; Imamura et al., 2014). Upregulated NEAT1-2 would induce SPFQ sequestration into paraspeckles, thus removing the transcriptionally suppressive effects of SFPQ on the *ll-8* gene as well as other cytokine genes, and strengthening host inflammatory responses (Imamura et al., 2014). NEAT1-2 could also interact with and enhance the assembly of inflammasome components in macrophages, which would accelerate subsequent pro-caspase-1 processing and promote IL-1β production (Zhang et al., 2019). We previously demonstrated that NEAT1-2 could be activated by HTNV through the RIG-I-IRF7 pathway and in turn positively regulate RIG-I-mediated type I IFN responses in endovascular cells (Ma H. et al., 2017), while it is unclear what role NEAT1 plays in HTNV-stimulated macrophage activation and how NEAT1 is associated with disease progression.

In this study, we examined the gene expression profile of macrophages at different time points after HTNV infection through high-throughput RNA sequencing (RNA-seq) and observed elevated NEAT1 expression, as well as the enhanced cholesterol synthesis process, at the early stage. Interfering with NEAT1-2 transcription could restrain inflammatory macrophage activation and thus facilitate HTNV propagation in a macrophage-endovascular coculture system, while forced expression of NEAT1-2 stimulated macrophages to secrete various cytokines and chemokines, such as TNFα and IFNα, hence restricting HTNV infection. Mechanistically, we found that NEAT1-2 might upregulate *Srebf2* expression, bind to SREBP2, and positively influence its proinflammatory activity. Moreover, the NEAT1-2 transcription level in monocytes was associated with viral load and disease severity. These results reveal a previously unrecognized role of NEAT1 in metabolism and macrophage polarization and suggest that the altered expression of NEAT1-2 might be a potential biomarker for predicting HFRS progression.

## Materials and Methods

### Cell Culture and Transfection

HEK 293 T and Vero E6 cells were purchased from the China Center for Type Culture Collection (CCTCC), and cultured in Dulbecco’s modified Eagle’s medium (DMEM, HyClone) supplemented with 10% (v/v) fetal bovine serum (FBS, Gibco). HUVECs were purchased from ScienCell Research Laboratories and cocultured in endothelial cell medium (ECM) with endothelial cell growth supplement (ECGS) in a transwell system.

To generate murine bone marrow-derived macrophages (mBMDMs), the femur and tibia were removed from sacrificed adult mice. The bones were first rinsed with sterile phosphate-buffered saline (PBS) containing 0.1% (v/v) penicillin–streptomycin (P/S) solution. Subsequently, the bone marrow was flushed with Roswell Park Memorial Institute 1640 (RPMI 1640, HyClone) containing 10% FBS and 0.1% P/S and filtered with a cell strainer (70 mm). Cells were resuspended in RPMI 1640 after centrifugation and then primed with M-CSF (20 ng/ml, PeproTech) for 4 days, with medium exchange every other day, to generate mBMDMs.

To obtain human monocyte-derived macrophages (hMDMs), peripheral blood mononuclear cells (PBMCs) were first enriched from peripheral blood by Ficoll (TBDscience) density gradient centrifugation. Then, human monocytes (hMo) were magnetically purified from PBMCs with negative screening beads (EasySep™ Human Monocyte Isolation Kit, StemCell). Finally, monocytes were primed with recombinant human macrophage colony-stimulating factor (M-CSF; 15 ng/ml, PeproTech) for a week with medium exchange every other day to generate hMDMs. The primary mouse monocytes (mMo) were acquired through a protocol similar to that used for hMo.

The indicated plasmids were transfected into HEK 293T cells using JetPEI reagents (Polyplus). The siRNA transfection was performed with Lipofectamine 2000 (Invitrogen) at 24 h prior to infection; the sequences used for siRNA are shown in Table 1. The exogenous expression of plasmids in hMDMs relied on electrotransfection with the Neon™ transfection system instruments (Invitrogen, Cat# MPK5000). SFN (no. S4441) purchased from Sigma was added to the cells at a final concentration of 20 μM for short-term treatments up to 12 h and at a final concentration of 10 μM post HTNV infection.

**Table 1:** The sequences for qRT-PCR primers and siRNAs.

Gene Name	Primer sequences (5′-3′)
*Homo*-NEAT1-F	TTGTTCCAGAGCCCATGAT
*Homo*-NEAT1-R	TGAAAACCTTTACCCCAGGA
*Homo*-NEAT1-2-F	GATCTTTTCCACCCCAAGAGTACATAA
*Homo*-NEAT1-2-R	CTCACACAAACACAGATTCCACAAC
*Mus-*NEAT1-F	AGGAGAAGCGGGGCTAAGTA
*Mus-*NEAT1-R	TAGGACACTGCCCCCATGTA
*Mus*-NEAT1-2-F	CCTTGAGCCTGCAGACAAGA
*Mus*-NEAT1-2-R	ACTTACAGCCTTAACGCCCC
*Homo*-*Gapdh*-F	TGTGGGCATCAATGGATTTGG
*Homo*-*Gapdh*-R	ACACCATGTATTCCGGGTCAAT
*Mus*-*Gapdh*-F	AGGTCGGTGTGAACGGATTTG
*Mus*-*Gapdh*-R	TGTAGACCATGTAGTTGAGGTCA
HTNV-S-F	TCTAGTTGTATCCCCATCGACTG
HTNV-S-R	ACATGCGGAATACAATTATGGC
*Homo*-*Tnfα*-F	CCTCTCTCTAATCAGCCCTCTG
*Homo*-*Tnfα*-R	GAGGACCTGGGAGTAGATGAG
*Homo*-*Ifnα*-F	GCCTCGCCCTTTGCTTTACT
*Homo*-*Ifnα*-R	CTGTGGGTCTCAGGGAGATCA
*Homo*-*Il-1β*-F	AGCTACGAATCTCCGACCAC
*Homo*-*Il-1β*-R	CGTTATCCCATGTGTCGAAGAA
*Homo*-*Il-8*-F	AGACAGCAGAGCACACAAGC
*Homo*-*Il-8*-R	ATGGTTCCTTCCGGTGGT
*Homo*-*Il-12*-F	ACCCTGACCATCCAAGTCAAA
*Homo-Il-12*-R	TTGGCCTCGCATCTTAGAAAG
*Homo*-*Cd80*-F	GGGCACATACGAGTGTGTTGT
*Homo-Cd80*-R	TCAGCTTTGACTGATAACGTCAC
*Homo*-*Ccl2*-F	CAGCCAGATGCAATCAATGCC
*Homo*-*Ccl2*-R	TGGAATCCTGAACCCACTTCT
*Homo*-*Ccl5*-F	CCAGCAGTCGTCTTTGTCAC
*Homo*-*Ccl5*-R	CTCTGGGTTGGCACACACTT
*Homo*-*Cxcl10*-F	GTGGCATTCAAGGAGTACCTC
*Homo*-*Cxcl10*-R	TGATGGCCTTCGATTCTGGATT
*Homo*-*Il-10*-F	GACTTTAAGGGTTACCTGGGTTG
*Homo*-*Il-10*-R	TCACATGCGCCTTGATGTCTG
*Homo*-*Tgfβ*-F	GTAGCTCTGATGAGTGCAATGAC
*Homo*-*Tgfβ*-R	CAGATATGGCAACTCCCAGTG
*Homo*-*Cd206*-F	TCCGGGTGCTGTTCTCCTA
*Homo*-*Cd206*-R	CCAGTCTGTTTTTGATGGCACT
*Homo*-*Srebf1*-F	CGGAACCATCTTGGCAACAGT
*Homo*- *Srebf1*-R	CGCTTCTCAATGGCGTTGT
*Homo*- *Srebf2*-F	CTGCAACAACAGACGGTAATGA
*Homo*- *Srebf2*-R	CCATTGGCCGTTTGTGTCAG
*Homo*-*Dhcr7*-F	CACTGGCGAGCGTCATCTT
*Homo*-*Dhcr7*-R	TCCTCGTTATAGGTGGAGTCTTG
*Homo*- *Dhcr24*-F	GCACAGGCATCGAGTCATCAT
*Homo*- *Dhcr24*-R	GTGCATCGCACAAAGCTGC
*Homo*-*Sqle*-F	GGCATTGCCACTTTCACCTAT
*Homo*-*Sqle*-R	GGUCTGAGAGAATATCCGAGAAG
si-NC-sense	AAUAAGGUUCUUAGUUAGACGUGACUG
si-NEAT1-2-sense	GUGAGAAGUUGCUUAGAAACUUUCC
si-BF1-sense	GUTGATGGATGTGUTGAUTAGTGAGAGT
si-BF2-sense	GAGGUTGAGTTGUTGTAGUGTUTTGA

### RNA-seq Analysis

Total RNA was extracted from mBMDMs at 0, 12, 24, or 36 hpi using TRIzol (Invitrogen). Ribosomal RNA was removed using the Ribo-Zero™ kit (Epicenter Biotechnologies). Fragmented RNA (average length approximately 200 bp) was subjected to first-strand and second-strand cDNA synthesis followed by adaptor ligation and enrichment with a low-cycle number PCR. The libraries were paired-end sequenced at Guangzhou Ribo Biotechnology (Guangzhou, China) using the Illumina HiSeq 3000 platform. The expression values [reads per kilobase million (RPKM)] were normalized per gene over all samples, the mean and standard deviation (SD) of expression over all samples were calculated for each gene, and the expression value was linearly transformed using the formula (RPKM-mean)/SD. The KEGG, GO, and time-sequenced gene analyses were performed using the Omicshare platform.^1^ RNA-seq data are available online from the ArrayExpress database under accession number E-MTAB-11353.

### Viral Propagation and Evaluation

The HTNV strain used in this paper (76-118) was maintained in our laboratory and propagated in Vero E6 cells. Cultured cells were infected with HTNV at the indicated multiplicities of infection (MOIs). After 2 h of incubation of cells with HTNV, the virus-containing medium was discarded, and the cells were washed thoroughly with sterile medium and then resuspended in culture medium. As a control, cells were incubated with culture supernatant from uninfected Vero E6 cells or cells infected with Co^60^ irradiation-inactivated HTNV. The viral load and viral titer were detected as we previously described (Ma H. et al., 2017; Ma H. W. et al., 2017; Ye et al., 2019).

### RNA-Associated Measurements

#### Quantitative Real-Time PCR

Total cellular RNA was extracted with TRIzol reagent (Invitrogen) and the Total RNA Extraction Kit (TIANGEN Biotech), and the concentration of the extracted RNA was measured with a spectrophotometer. Quantitative real-time PCR (qRT-PCR) was performed with PrimeScript RT Master Mix (TaKaRa) according to the manufacturer’s protocol. Each cDNA sample was denatured at 95°C for 5 min and amplified for 35 cycles of 15 s at 98°C, 30 s at 58°C, and 30 s at 72°C with a LightCycler 96 (Roche). The mRNA expression level of each target gene was normalized to GAPDH and analyzed using LightCycler® 96 Application Software (Roche). The qRT-PCR primers are listed in Table 1.

#### RNAScope

RNAScope was performed with an RNAscope Fluorescent Multiplex Reagent Kit (ACD Bio) based on the manufacturer’s protocols. In short, cells were first placed on slides and fixed in 4% PFA (Sigma–Aldrich) for 30 min, followed by antigen repair with RNAscope® hydrogen peroxide (ACD Bio) for 10 min at room temperature (RT) and digestion with RNAscope® protease III (ACD Bio) for another 10 min at RT in a humid chamber. Next, the cells were incubated at 40°C with the following solutions: (1) RNAScope probes of target RNAs, namely, lncRNA NEAT1-2, in hybridization buffer A for 2 h; (2) preamplifier in hybridization buffer B for 30 min; (3) amplifier in hybridization buffer B at 40°C for 30 min; and (4) label probe in hybridization buffer C for 15 min. After each hybridization step, the slides were washed with wash buffer three times at RT. Then, the probe signal was further recognized and amplified by HRP-C2, followed by chromogenic detection with TSA® Plus Cy5 for detecting lncRNA NEAT1-2. Finally, after DAPI staining and Prolong Gold Antifade Mountant (Thermo Fisher, P36930) treatment, the samples were observed with a confocal microscope (Nikon).

#### RIP Assays

RIP assays were performed using a Magna RIP™ RNA-Binding Protein Immunoprecipitation Kit (Millipore, 17-700) according to the manufacturer’s instructions in an RNase-free environment. Briefly, HEK 293T cells were transfected with plasmids encoding flag-Stat1 (Sino Biological, HG12766-NF), flag-p65 (Sino Biological, HG12054-NF), flag-SREBP1 (Sino Biological, HG17512-*CF*), or flag-SREBP2 (OriGene, RC208942) for 24 h and infected with HTNV at an MOI of 5. Cells at various time points after HTNV infection were collected and treated with RIP Lysis Buffer. The anti-flag antibody-conjugated magnetic beads (Bimake, B26102) were incubated with cell lysates on a shaker overnight at 4°C. For the positive control, the anti-SNRNP70 antibody, which pulls down the U1 snRNA, was applied. The supernatants were discarded after washing on the magnetic frame; then, proteinase K was added to the pellets with gentle shaking for 30 min at 55°C. Finally, the supernatants were collected on the magnetic frame, from which the total target protein-attached RNAs were extracted as mentioned above. The enriched RNAs were detected by qRT-PCR and normalized to the positive control (U1 snRNA).

### Protein-Related Measurements

#### Immunoblot Assays

The protein concentration was first determined based on the bicinchoninic acid (BCA) method using the Compat-Able™ BCA Protein Assay Kit (Thermo Fisher, 21063). Equal amounts of protein were boiled at 95°C for 10 min, separated by SDS–PAGE with a 10% gel (running at 120 V for 120 min), and then electrophoretically transferred onto polyvinylidene fluoride membranes (PVDF; transferred at 100 V for 100 min). After blocking with 5% nonfat milk in TBS, the membrane was incubated with the primary antibodies, followed by secondary antibodies labeled with infrared dyes. The primary antibodies used in this paper were anti-HTNV NP 1A8 mouse monoclonal antibody (constructed and maintained by our laboratory; Ma H. et al., 2017), anti-IFITM3 rabbit polyclonal antibody (Proteintech, 11714-1-AP), anti-Mx1 rabbit polyclonal antibody (Proteintech, 13750-1-AP), anti-Stat1 rabbit monoclonal antibody (Abcam, ab234400), anti-Stat1(phospho S727) rabbit monoclonal antibody (Abcam, ab109461), anti-p65 rabbit monoclonal antibody (Abcam, ab32536), anti-p65(phospho S536) rabbit monoclonal antibody (Abcam, ab32536), anti-SREBP1 rabbit polyclonal antibody (Abcam, ab28481), anti-SREBP2 rabbit polyclonal antibody (Abcam, ab30682), anti-SFPQ rabbit polyclonal antibody (Proteintech, 15585-1-AP), anti-NONO rabbit polyclonal antibody (Proteintech, 11058-1-AP), anti-flag rabbit monoclonal antibody (Sigma, F2555), and anti-GAPDH mouse monoclonal antibody (Proteintech, 60004-1-Ig). The secondary antibodies used in this paper were 680RD goat anti-mouse IgG (LI-COR, 925-68070) and 800CW goat anti-rabbit IgG (LI-COR, 926-32211). The signals on the PVDF membrane were visualized using the Odyssey Infrared Imaging System (LI-COR Biosciences).

#### Immunofluorescence Assays

Cells exposed to the indicated treatment were fixed with ice-cold 4% (w/v) paraformaldehyde (PFA, Sigma–Aldrich) for 15 min and then permeabilized with 0.1% Triton X-100 (Sigma–Aldrich) for 20 min at RT. After blocking with 3% bovine serum albumin (BSA, Sigma–Aldrich) for 30 min, specific primary antibodies, namely, anti-HTNV NP 1A8 mouse monoclonal antibody (prepared and maintained by our laboratory; 1:50 dilution) and anti-SREBP2 rabbit polyclonal antibody (Abcam, ab30682; 1:50 dilution), were added and incubated overnight at 4°C. After five washes with DPBS, the secondary antibodies, namely, FITC-conjugated goat anti-mouse IgG (Abcam, ab6785) and Cy3-conjugated goat anti-rabbit IgG (Abcam, ab6939), were used for detection (incubation at 37°C for 1 h). Cell nuclei were stained with DAPI (Thermo Fisher, D9542) for 5 min at RT. After sealing with ProLong™ Gold Antifade Mountant (Thermo Fisher, P36930), the samples were observed using a fluorescence microscope (A1R-HD25, Nikon).

#### Flow Cytometry Assays

Generally, the FcγII/III receptors of macrophages were blocked with anti-CD16/32 antibody (BD Bioscience) before staining the cell surface markers, and brilliant stain buffer (BD Bioscience) was applied prior to staining intracellular cytokines. The main procedure was as follows: Cells were acquired, and Fc receptor block was performed, followed by surface marker staining, permeabilization and fixation. Then, the cells were treated with brilliant stain buffer, followed by intracellular cytokine or HTNV NP staining. Compensation adjustment with beads was carried out, and then the samples were analyzed with a 3-laser BD FACSCalibur flow cytometer. Finally, the data were processed with FlowJo v10 (TreeStar). The flow cytometry assay-related antibodies included PerCP-Cy™5.5 mouse anti-human TNF (BD Biosciences, 60679), BV421 rat anti-human and viral IL-10 (BD Biosciences, 564053) and FITC mouse anti-HTNV NP (constructed and maintained in our laboratory).

### Cytokine Concentration Detection

#### Enzyme-Linked Immunosorbent Assay

The concentrations of IL-8 and CCL5 in cell supernatants or mouse tissues were evaluated with ELISA kits (Abcam or R&D Systems) according to the manufacturer’s instructions. In short, standard samples were prepared with gradient dilution to build the standard curve. The samples were diluted with specific buffer, added to a plate precoated with anti-cytokine or chemokine antibodies, and then reacted with HRP-conjugated detection antibodies. TMB and stop solution were added sequentially, and then the OD450 was measured. The cytokine or chemokine concentration was calculated according to the standard curve.

#### BioPlex Multiplex Immunoassays

The serum or cell supernatant samples were centrifuged at 10,000 rpm for 15 min at 4°C and then diluted (for serum, the sample diluent was HB, 1:4; for cell supernatants, the diluent was culture medium, 1:5). After preparing the standards, controls and samples, the BioPlex Multiplex Immunoassays were conducted according to the following workflow. The cells were prewetted, and the magnetic beads containing the antibodies against various cytokines and chemokines (50 μl, 40 kinds of cytokines and chemokines) were added. The sample/standard/control were added and incubated with shaking at 850 rpm for 1 h at RT, followed by the addition of biotinylated detection antibodies containing the phycoerythrin fluorescent reporters (25 μl, incubation with shaking at 850 rpm for 30 min at RT). Then, streptavidin-PE was added (50 μl, incubation on shaker at 850 rpm for 10 min at RT), and the beads were resuspended in assay buffer (125 μl, shaking at 850 rpm for 30 s). Finally, data were acquired on a Bio-Plex system (Bio–Rad).

### Transcription Factor Activity Detection

The transcriptional activities of Stat1 (Abcam, ab207228), p65 (Abcam, ab133112), SREBP1 (Abcam, ab133125), and SREBP2 (Abcam, ab133111) were detected with the corresponding kits according to the manufacturer’s instructions. In brief, hMDMs subjected to the desired treatments after HTNV infection were collected for extraction of the nuclear protein, which contained multiple activated transcription factors. Then, the nuclear extract was added to a 96-well plate containing the Stat1, p65, SREBP1, or SREBP2 response elements (double-stranded DNA sequence). The corresponding transcription factor was detected by the addition of a specific primary antibody directed against it. A secondary antibody conjugated to HRP was added to provide a sensitive colorimetric readout at 450 nm.

### ROS Measurements

Cellular ROS production was detected with DCFDA/H2DCFDA. In brief, mBMDMs subjected to the indicated treatments were harvested and seeded into a dark, clear-bottomed 96-well microplate and stained by incubating with DCFDA Solution (100 μl/well) for 45 min at 37°C in the dark. The plate was analyzed immediately on a fluorescence plate reader at Ex/Em = 485/535 nm in end point mode.

### Study Participants

This study included 123 blood samples from 85 HFRS patients hospitalized in the Department of Infectious Diseases at the Tangdu Hospital of the Fourth Military Medical University (Xi’an, China) from December 2016 to December 2019. All samples were collected before dialysis treatment or continuous renal replacement therapy. HTNV infection was diagnosed by serological testing according to diagnostic criteria. To control for potential confounders, we excluded patients with autoimmune diseases, viral hepatitis, hematological diseases, diabetes, cardiovascular diseases, and other kidney or liver diseases. Basic clinical data are shown in Table 2. The criteria for disease course and severity classification have been described (Ma et al., 2015; Ma H. W. et al., 2017). For the control group, fresh peripheral blood was obtained from 30 healthy donors for PBMC isolation. Before blood collection, all HFRS patients and healthy donors provided informed and signed written consent to participation in the study.

**Table 2:** Clinical and laboratory features of patients with HFRS stratified by five stages.

	Acute phase			Convalescent phase	
	Febrile (*n* = 24)	Hypotensive (*n* = 24)	Oliguric (*n* = 34)	Diuretic (*n* = 24)	Convalescent (*n* = 17)
Age (years)	42 [37, 45]	44 [34, 52]	41 [32, 47]	45 [38, 50]	48 [41, 57]
Gender (male)	9 (37.5%)	13 (54.2%)	18 (52.9%)	14 (58.3%)	10 (58.8%)
Gender (female)	15 (62.5%)	11 (45.8%)	16 (47.1%)	10 (41.7%)	7 (41.2%)
WBC (×10^9^/L)	8.7 [6.6, 11.5]	10.2 [6.7, 13.5]	12.0 [8.3, 15.5]	6.5 [4.9, 7.9]	7.9 [6.8, 9.2]
Platelet count (10^12^/L)	109.5 [75.4, 288.9]	78.5 [42.6, 134.5]	165.0 [114.0, 212.9]	191.0 [151.1, 217]	214.4 [159.9, 219.1]
Hemoglobin (g/L)	123.5 [103.4, 136.8]	128.5 [97.1, 152.8]	118.5 [109.2, 137.4]	111.4 [98.4, 120.4]	122.4 [103.1, 134.8]
Monocyte count (×10^9^/L)	1.4 [1.1, 1.9]	1.6 [1.3, 2.0]	1.5 [0.6, 2.2]	0.7 [0.5, 0.8]	0.4 [0.3, 0.7]
Neutrophil count (×10^9^/L)	6.7 [5.8, 8.2]	6.1 [5.2, 7.6]	7.2 [4.5, 8.4]	4.3 [2.4, 5.5]	4.4 [2.9, 5.6]
Lymphocyte count (×10^9^/L)	3.8 [3.1, 4.6]	2.45 [1.3, 3.8]	2.4 [1.7, 4.1]	1.9 [1.5, 2.3]	2.2 [1.7, 2.6]
Serum creatinine (μmol/L)	169.3 [101.8, 266.3]	234 [142.5, 455]	306.1 [250.2, 416.6]	193.1 [178.4, 212.5]	71.8 [62.9,83.2]
Urea nitrogen (mmol/L)	11.7 [9.1, 13.0]	11.95 [11.2, 14.9]	13 [11.1, 15]	12.9 [8.9,14.9]	7.8 [7.0,8.8]

Data are presented as the median (interquartile range, IQR) or *n* (%).

### Statistical Analysis

Statistical analysis was performed with GraphPad Prism (GraphPad software, version 8). For the comparison of two groups, two-tailed unpaired Student’s *t*-test was applied, unless stated otherwise in the figure legend. For multiple comparisons, one-way ANOVA was performed. Correlations among the investigated traits are represented by Spearman’s rank correlation coefficients. Differences were considered to be statistically significant when the values of *p* were < 0.05 (*), <0.01 (**), or < 0.001 (***). Data are presented as the mean ± SEM if not stated otherwise in the figure legend. The number of independent experiments conducted is shown in the figure legend.

## Results

### NEAT1-2 Expression Is Elevated at the Early HTNV Infection Phase in Monocytes and Macrophages

**Figure 1:**
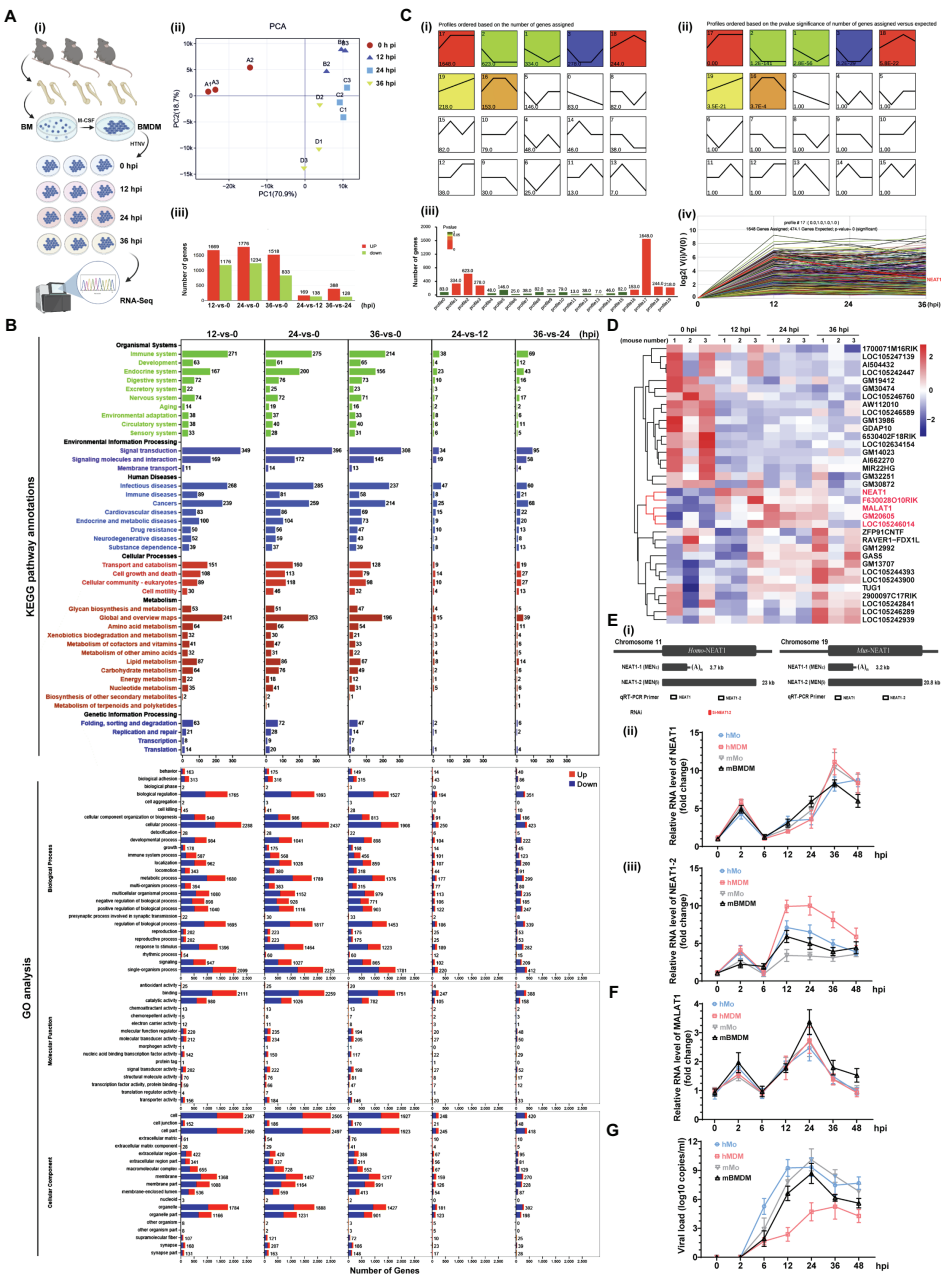
RNA-seq analysis of HTNV-infected macrophages and lncRNA identification. **(A) (i)** Experimental design for RNA-seq. **(ii)** PCA of different groups. **(iii)** Analysis of differentially expressed genes between groups. **(B)** The KEGG pathway annotations (upper) and GO analysis (bottom) of RNA-seq data from A. **(C) (i)** List FIGURE 1 | of time-sequenced gene expression profiles ordered based on the number of genes assigned. **(ii)** List of time-sequenced gene expression profiles ordered based on the significance (value of *p*) of the number of genes assigned vs. expected. **(iii)** The number of genes and related values of *p* from profile 0 to 19. **(iv)** The differentially expressed genes in profile 17. **(D)** Heatmap of differentially expressed lncRNAs from 0 to 36 hpi. **(E) (i)** The target positions of primers or siRNAs designed for qRT-PCR or RNA interference (RNAi). **(ii)** qRT-PCR analysis of NEAT1 in primary human monocytes (hMo), hMo-derived macrophages (hMDMs), mouse monocytes (mMo), and mouse bone marrow-derived macrophages (mBMDMs) from 0 to 48 hpi with an MOI of 5. **(ii)** qRT-PCR analysis of NEAT1-2 from **(i)** (*n* = 5 in each group). **(F)** qRT-PCR analysis of MALAT1 from **E-(i)**. **(G)** The viral load calculated by qRT-PCR from **E-(i)**. Data are shown as the mean ± SEM and are representative of three independent experiments.

We analyzed the HTNV-stimulated responses using RNA-seq of mouse bone marrow-derived macrophages (mBMDMs) that were infected with HTNV from 0 to 36 h postinfection (hpi; Figure 1A-i). Principal component analysis (PCA) indicated that the three replicates within groups had low variation, and it seemed that infected groups (from 12 to 36 hpi) tended to present similar gene expression profiles in comparison with the mock-infected group (Figure 1A-ii). Accordingly, compared with the 0 hpi group, 1,000 of genes were differentially expressed at 12, 24, or 36 hpi, while fewer genes were significantly changed between 24 and 12 hpi or 36 and 24 hpi (Figure 1A-iii). Kyoto Encyclopedia of Genes and Genomes (KEGG) pathway enrichment analysis revealed that most immune genes associated with infectious diseases were activated after HTNV infection, and notably, the expression of metabolism-related genes, especially those in lipid metabolic pathways, showed a significant alteration (Figure 1B, upper). Gene Ontology (GO) analysis showed that the proportion of upregulated genes (shown in red) generally decreased from 12 to 36 hpi, indicating that the acute alteration of gene expression might play an important antiviral role that would be subverted along with HTNV replication (Figure 1B, bottom). The number of metabolic process-associated genes exceeded that of immune process-associated genes, further suggesting the importance of metabolism after HTNV infection (Figure 1B, bottom). To define the gene expression trends at various time points, the profiles were summarized with the number of genes assigned or the statistical significance (Figures 1C-i,ii), of which profile 17, which increased first and then remained continuously elevated, harbored the most genes (1,648 genes; Figures 1C-iii,iv). Among the profile 17 gene group, we found five previously reported lncRNAs, of which NEAT1 showed the highest alteration ratio at the early phase (from 0 to 24 hpi) with the highest endogenous expression (ranging from 134.797 to 224.733 reads per kilobase per million mapped reads/RPKM; Figure 1C-iv,D, shown in red). To verify NEAT1 alteration in primary human and mouse macrophages or monocytes, different primers targeting NEAT1 (both NEAT1-1 and NEAT1-2) or NEAT1-2 only were designed for quantitative real-time polymerase chain reaction (qRT-PCR; Figure 1E-i). We found that the expression of NEAT1, especially NEAT1-2, was obviously induced from 12 to 36 hpi in both primary human and mouse monocytes and macrophages (Figures 1E-ii,iii). As a control, lncRNA metastasis-associated lung adenocarcinoma transcript 1 (MALAT1), the vital component for speckle structure in the nucleus that might participate in the viral life cycle (Swain et al., 2021), was detected, and the data showed that MALAT1 expression increased from 0 to 24 hpi and then collapsed (Figure 1F). Intriguingly, the transcription level of NEAT1-2 seemed to persistently exhibit higher fold changes in macrophages than in monocytes (Figure 1E-iii), and macrophages maintained a relatively lower viral load of HTNV than their counterpart monocytes (Figure 1G). These results suggested that lncRNA NEAT1 was induced in monocytes and macrophages at the early HTNV infection phase and exhibited expression level changes similar to those of most of the other differentially expressed genes.

### NEAT1-2 Stimulates Inflammatory Macrophage Activation to Limit HTNV Propagation

**Figure 2:**
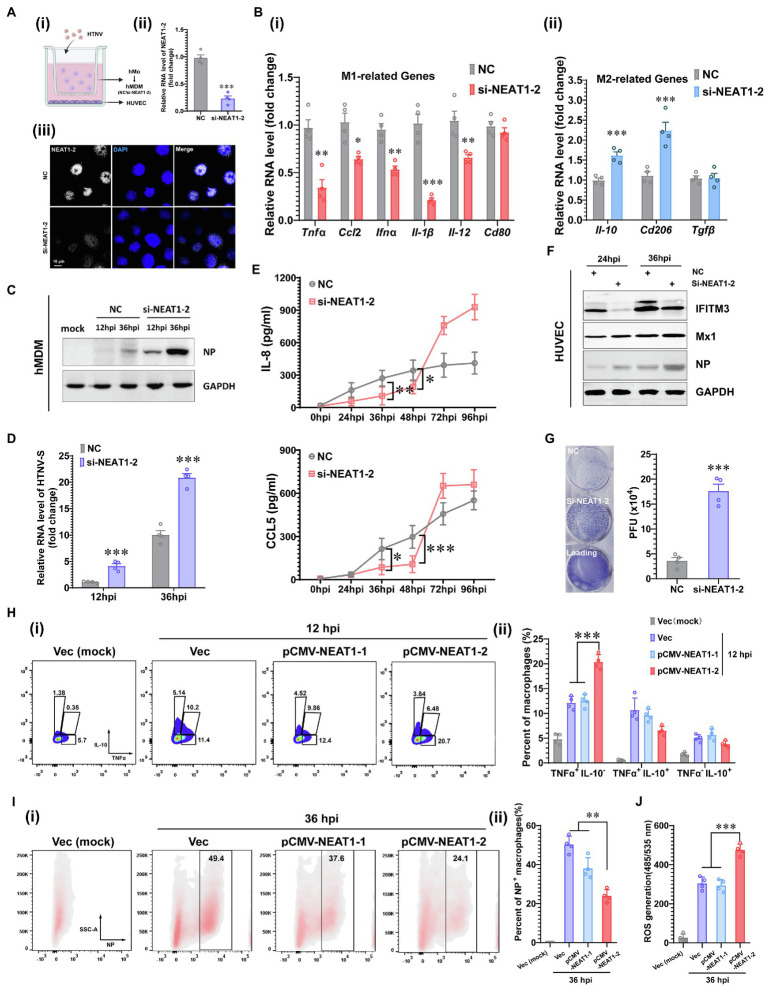
Enhanced M1 Polarization by NEAT1-2 to Constrain HTNV Propagation. **(A) (i)** Schematic diagram of the coculture system. **(ii)** RNAi efficiency of NEAT1-2 silencing in hMDMs confirmed by qRT-PCR. **(iii)** RNAi efficiency of NEAT1-2 in hMDMs confirmed by RNAScope. NC, negative control with scrambled RNAs. **(B) (i)** qRT-PCR analysis of M1-related genes in hMDMs from the coculture system shown in **2A-(i)**. **(ii)** qRT-PCR analysis of M2-related genes in hMDMs from FIGURE 2 | the coculture system. **(C)** Immunoblot analysis of HTNV NPs in hMDMs from the coculture system. **(D)** qRT-PCR analysis of HTNV S segment hMDMs from the coculture system. **(E)** Enzyme-linked immunosorbent assay (ELISA) detection of IL-8 and CCL5 concentrations in the bottom medium in the coculture system. **(F)** Immunoblot analysis of the indicated proteins in HUVECs in the coculture system. **(G)** Viral titers of HUVECs assessed by the improved enzyme-linked focus formation assays. **(H) (i)** Representative flow cytometry data for TNFα and IL-10 production in NEAT1- or NEAT1-2-overexpressing hMDMs at 12 hpi (MOI = 5). **(ii)** Statistical analysis of data from **(i)**. **(I)** **(i)** Representative flow cytometry data for HTNV NP expression in NEAT1- or NEAT1-2-overexpressing hMDMs at 36 hpi (MOI = 5). **(ii)** Statistical analysis of data from **(i)**. **(J)** ROS measurement of hMDMs from **I-(i)**. Data are shown as the mean ± SEM and are representative of three independent experiments. Each point represents a single sample (*n* = 4 in each group). Analysis was performed using the unpaired Student’s *t*-test **(A–G)** or one-way ANOVA **(H–J)**. *p < 0.05, **p < 0.01, and ***p < 0.001.

To evaluate whether NEAT1-2 upregulation affects the macrophage activation pattern and viral propagation, silencing experiments were performed in a coculture system in which human umbilical vein endothelial cells (HUVECs) and monocyte-derived macrophages (hMDMs) were inoculated into the bottom or middle layer of a transwell system (Figure 2A-i). To specifically investigate the effects of NEAT1-2 on macrophages, hMDMs were independently transfected with small interfering RNAs (siRNAs) designed exclusively to target NEAT1-2 (Figure 1E-i), and the ablation efficiency of NEAT1-2 in hMDMs was confirmed by qRT-PCR and RNAScope at 24 h post transfection (Figure 2A-ii,iii). Then, NEAT1-2-silenced hMDMs were cocultured with HUVECs for another 24 h before HTNV was added to the upper medium with an MOI of 5 for hMDMs (Figure 2A-i). We found that knocking down NEAT1-2 could remarkably subvert macrophage identity by inhibiting the transcription of many M1-related genes, such as the proinflammatory *Tnfα*, the chemokine *Ccl5* and the antiviral *Ifnα* (Figure 2B-i), but not the antigen presenting-associated *Cd80*, and selectively upregulated the expression of M2-related genes, including *Il-10* and *Cd206* (Figure 2B-i). The replicative efficiency of HTNV was evaluated at the protein (NP) and RNA (HTNV-S) levels, and the results suggested that the loss of function of NEAT1-2 distinctively facilitated viral replication (Figures 2C,D). Furthermore, the crosstalk between the middle hMDMs and the bottom HUVECs was detected. The IL-8 and CCL5 concentrations in the bottom medium from 26 hpi to 48 hpi were greatly reduced in the group with NEAT1-2-silenced hMDMs compared with the control group (Figure 2E), and the bottom HUVECs also seemed to be more sensitive to HTNV infection (Figure 2F). Notably, the expression of the interferon-stimulated gene IFITM3, which we previously showed to suppress HTNV entry (Xu-Yang et al., 2016; Ma H. W. et al., 2017), but not Mx1, was delayed in HUVECs that were cocultured with NEAT1-2-silenced hMDMs (Figure 2F), which might partially explain why they were susceptible to HTNV infection. Next, the viral titers were detected with the improved enzyme-linked focus formation assay that we previously established (Ye et al., 2019), and the results indicated that progeny virus production was increased in HUVECs that were cocultured with NEAT1-2-silenced hMDMs (Figure 2G). To exclude the effects of NEAT1-1 on macrophage activation, different plasmids expressing either NEAT1-1 or NEAT1-2 were exploited as we previously reported (Ma H. et al., 2017). We found that overexpression of NEAT1-2, but not NEAT1-1, promoted TNFα release (Figure 2H) and restricted HTNV replication (Figure 2I) in primary macrophages. Additionally, the enhancement of NEAT1-2 expression strengthened ROS production, indicating that the antiviral and bactericidal activity in macrophages was improved after HTNV infection (Figure 2J). Previous study indicated that sulforaphane (SFN) induced both NEAT1-1 and NEAT1-2 expression, so we wondered whether SFN treatment could restrain HTNV infection in macrophages (Lellahi et al., 2018). As expected, we also found that NEAT1 expression was upregulated at different time points post SFN stimulation in mBMDMs (Supplementary Figure S1A). Pretreatment with SFN for 4 h inhibited HTNV replication (Supplementary Figure S1B), which was on a dose-dependent manner mBMDMs (Supplementary Figure S1C). Collectively, these data suggested that NEAT1-2 could positively influence M1 polarization, thus limiting the replication and propagation of HTNV.

### SREBP2-Mediated Cholesterol Synthesis Upon HTNV Infection Is Manipulated by NEAT1-2

**Figure 3:**
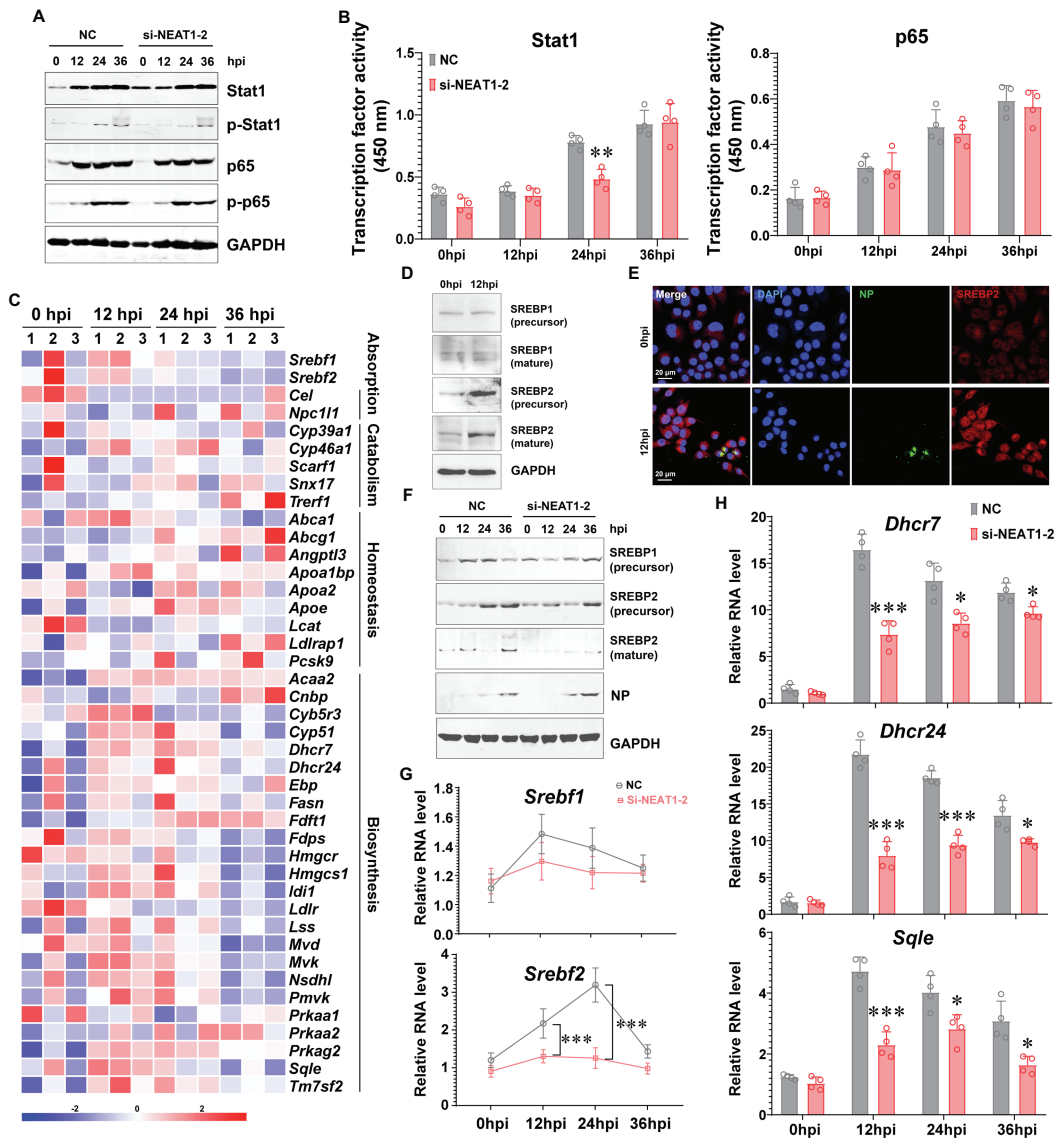
Regulation of the SREBP2 Pathway by NEAT1-2 after HTNV Infection. **(A)** Immunoblot analysis of total and phosphorylated Stat1/p65 in hMDMs treated with RNAi (MOI = 5). **(B)** Detection of the transcriptional activity of Stat1 and p65 in hMDMs from **(A)**. **(C)** Heatmap of genes involved in cholesterol metabolism of mBMDMs from Figure 1A. **(D)** Immunoblot analysis of the indicated proteins in hMDMs at 12 hpi with an MOI of 5. **(E)** Immunofluorescence assays for SREBP2 and HTNV NP in hMDMs at 12 hpi with an MOI of 5. **(F)** Immunoblot analysis of the indicated proteins in hMDMs treated with RNAi (MOI = 5). **(G)** qRT-PCR analysis of *Srebf1* and *Srebf2* in hMDMs from **(F)**. **(H)** qRT-PCR analysis of the indicated genes associated with cholesterol synthesis from **(F)**. Data are shown as the mean ± SEM and are representative of three independent experiments. Each point represents a single sample (*n* = 4 in each group). Analysis was performed using the unpaired Student’s *t*-test. *p < 0.05, **p < 0.01, and ***p < 0.001.

To interpret how NENAT1-2 regulates macrophage activation, the classical pathways involved in M1 polarization were evaluated. Unexpectedly, dysfunction of NEAT1-2 in hMDMs after HTNV infection scarcely affected the total expression or phosphorylation of Stat1 and p65 (Figure 3A), the transcriptional activity of which remained almost unchanged (Figure 3B). This implied that NEAT1-2 might modulate HTNV-induced macrophage activation through mechanisms other than the canonical JAK/STAT and NF-κB pathways. Considering that the RNA-seq data showed that the lipid metabolic pathway was enriched during HTNV infection (Figure 1B), we wondered whether NEAT1-2 could affect the metabolic reprogramming process of macrophages. To answer this question, we first analyzed the alteration of the sterol metabolism process after HTNV infection and found that most genes promoting cholesterol biosynthesis, other than those regulating cholesterol absorption, catabolism or homeostasis, were activated at the early phase (12 hpi; Figure 3C). Then, the expression and maturation of crucial transcription factors controlling lipid metabolism were investigated, and the results showed that SREBP2, instead of SREBP1, was remarkably stimulated (Figure 3D). Immunofluorescence assays (IFAs) also revealed that SREBP2 was translocated into the nucleus from 0 to 12 hpi, confirming its enhanced activity (Figure 3E). Next, we evaluated whether NEAT1-2 could regulate SREBP2 activation. NEAT1-2 ablation negatively affected HTNV-triggered expression of SREBP2 both at the transcriptional and posttranscriptional levels (Figures 3F,G); concomitantly, SREBP2 maturation was reduced, and the replication of HTNV was increased (Figure 3F). In contrast, NEAT1-2 knockdown did not affect SREBP1 gene expression from 0 to 36 hpi (Figure 3G). Furthermore, the target genes of SREBP2, such as *Dhcr7*, *Dhcr24*, and *Sqle*, were also downregulated under NEAT1-2 silencing conditions (Figure 3H), validating the idea that NEAT1-2 could regulate sterol metabolism in HTNV-infected macrophages by enhancing SREBP2 activity.

### SREBP2 Directly Contributes to M1 Activation by Promoting Inflammatory Cytokine Production

**Figure 4:**
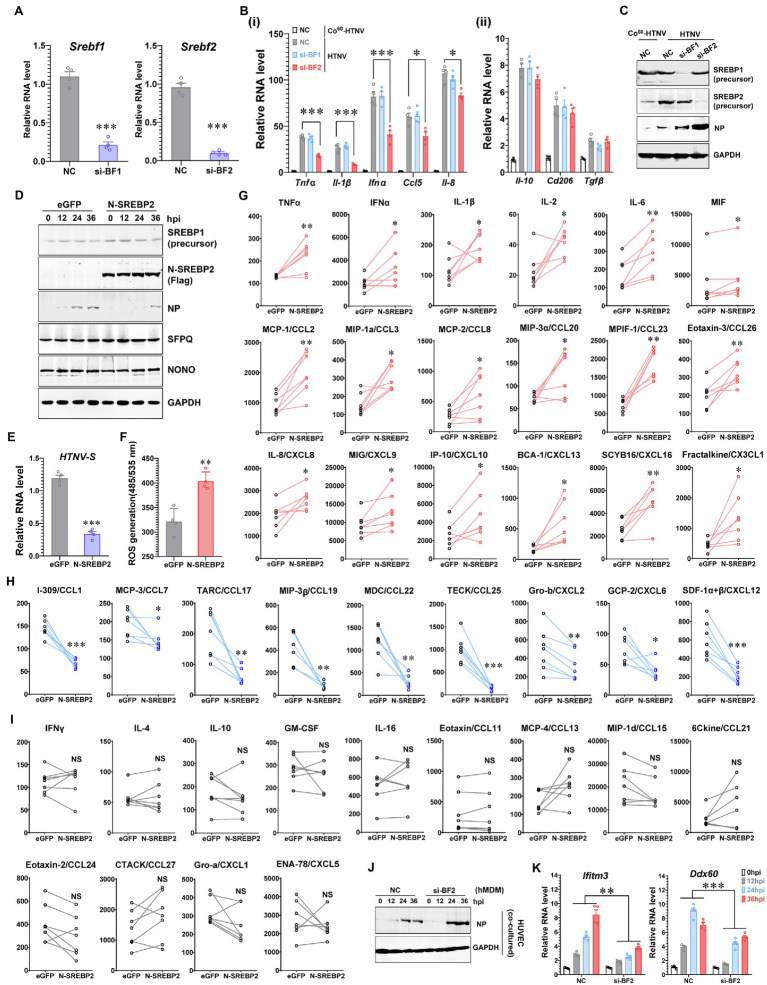
Promotion of Inflammatory Macrophage Phenotype by SREBP2 after HTNV Infection. **(A)** RNAi efficiency of silencing *Srebf1* (si-BF1) and *Srebf2* (si-BF2) in hMDMs confirmed by qRT-PCR. **(B) (i)** qRT-PCR analysis of M1-related genes in hMDMs at 24 hpi. **(ii)** qRT-PCR analysis of M2-related genes in hMDMs at 24 hpi. The hMDMs were transfected with siRNAs for 24 h and then infected with HTNV at an MOI of 5. **(C)** Immunoblot analysis of the indicated proteins in hMDMs from **(B)**. **(D)** Immunoblot analysis of the indicated proteins in hMDMs that were electrotransfected with plasmids expressing eGFP (as a control) or N-SREBP2 for 24 h and then infected with HTNV at an MOI of 5. **(E)** qRT-PCR analysis of HTNV S segments in hMDMs from **(D)**. **(F)** ROS detection in hMDMs from **(D)**. **(G)** Cytokines/chemokines upregulated by N-SREBP overexpression in HTNV-infected hMDMs at 36 hpi. The results were acquired through BioPlex Multiplex Immunoassays. The hMDMs were acquired and differentiated from seven healthy donors and then electrotransfected with the indicated plasmids for 24 h. The hMDMs from one donor were divided into two groups for the transfection of eGFP and N-SREBP2. Concentration (Y unit), pg/ml. **(H)** Downregulated cytokines/chemokines as in **(G)**. Concentration (Y unit), pg/ml. **(I)** Unchanged cytokines/chemokines as in **(G)**. Concentration (Y unit), pg/ml. **(J)** Immunoblot analysis of HTNV NPs in HUVECs. N-SREBP-overexpressing hMDMs were cocultured with HUVECs as designed in Figure 2A-i and then infected with HTNV at an MOI of 5. Immunoblot assays were performed at various time points after HTNV infection. **(K)** qRT-PCR analysis of the indicated genes in HUVECs from **(J)**. Data are shown as the mean ± SEM and are representative of three independent experiments. Each point represents a single sample (*n* = 4 in each group except **G–I**). Analysis was performed using the unpaired Student’s *t*-test **(A–F)**, paired Student’s *t*-test **(G–I)**, or one-way ANOVA **(K)**. *p < 0.05, **p < 0.01, and ***p < 0.001.

Increasing evidence indicates that SREBP2 directly modulates lipopolysaccharide (LPS)- or TNFα-induced immune responses (Guo et al., 2018; Kusnadi et al., 2019), and we wondered whether SREBP2 plays a role in HTNV-triggered inflammation in addition to its regulatory effects on the metabolic phenotype. We found that downregulating the expression of *Srebf2* (si-BF2), but not *Srebf1* (si-BF1), in hMDMs could impede the production of multiple M1-related cytokines at 24 hpi, including the inflammatory cytokines TNFα, IL-1β, and IFNα and the chemokines IL-8/CXCL8 and CCL5 (Figures 4A,B-i). Several M2-associated genes induced by HTNV infection, such as anti-inflammatory *Il-10* and *Tgfβ*, as well as the surface marker *Cd206*, remained unchanged under *Srebf1*- or *Srebf2*-silenced conditions (Figure 4B-ii). Accompanied by attenuated inflammation, HTNV replication increased with elevated NP production in *Srebf2*-silenced hMDMs (Figure 4C). These results indicated that SREBP2 might directly mediate M1 activation and restrain HTNV infection. To further confirm the regulatory role of SREBP2 in immune responses against HTNV infection, we overexpressed N-SREBP2, the transcriptionally active segment of SREBP2, and evaluated the resulting macrophage activation pattern as well as its antiviral ability. As expected, the overexpression of N-SREBP2 significantly inhibited HTNV replication in hMDMs from 12 to 36 hpi (Figures 4D,E), during which the ROS production was enhanced (Figure 4F), implicating that N-SREBP2 could stimulate the antimicrobial function of macrophages. Notably, upregulating N-SREBP2 did not affect the expression of SREBP1, which facilitates fatty acid synthesis, or the expression of NONO and SFPQ, which form the nuclear paraspeckle structure by interacting with NEAT1 (Figure 4D).

To illustrate the specific cytokines regulated by SREBP after HTNV infection, we measured the concentrations of 40 cytokines in the N-SREBP-overexpressing hMDMs (separated from seven independent heathy donors and cultured medium containing human serum) at 48 hpi with an MOI of 5 through Bio-Plex Multiplex Immunoassays (Figures 4G–I). The release of 18 cytokines, namely, TNFα, IFNα, IL-1β, IL-2, IL6, migration inhibitory factor (MIF), CCL2, CCL3, CCL8, CCL20, CCL23, CCL26, CXCL8, CXCL9, CXCL10, CXCL13, CXCL16, and CX3CL1, was enhanced in the N-SREBP exogenous expression group (Figure 4G), among which most had been reported to be elevated in HFRS patients (Mantula et al., 2018; Resman Rus et al., 2018). The production of another nine chemokines, including CCL1, CCL7, CCL17 and others, was decreased once N-SREBP was overexpressed (Figure 4H). However, the production of IFNγ, IL-4 and IL-10, which are canonical cytokines that stimulate M1 or M2 polarization (Wang et al., 2021), as well as 10 other kinds of cytokines, showed no significant conversion between the eGFP- and N-SREBP-overexpressing groups (Figure 4I). These data clarified that SREBP could promote the secretion of various inflammatory cytokines in macrophages to fight HTNV infection.

To elucidate whether SREBP-mediated inflammation could affect the intercellular propagation of HTNV, a coculture system was applied as designed in Figure 2A-i. Compared with that in the negative control (NC) group, HTNV NP production was elevated from 24 to 36 hpi in HUVECs cocultured with SREBP2-silenced hMDMs (si-BF2; Figure 4J), suggesting that SREBP2-mediated macrophage activation might restrict HTNV propagation in tissue cells. Another question was why HTNV showed differential replicative efficiency in HUVECs from the coculture system. We previously screened a series of host anti-hantaviral molecules and reported that IFITM3 and DDX60 exerted potent anti-HTNV effects in HUVECs (Xu-Yang et al., 2016; Ma H. W. et al., 2017). Here, we noted that macrophage dysfunction (caused by SREBP2 silencing) affected the expression of IFITM3 and DDX60 in HUVECs (Figure 4K), which might partially explain how hMDMs contributed to the antiviral status of HUVECs within the coculture system.

### NEAT1-2 Modulates M1 Polarization by Binding to SREBP2 and Enhancing Its Activity

**Figure 5:**
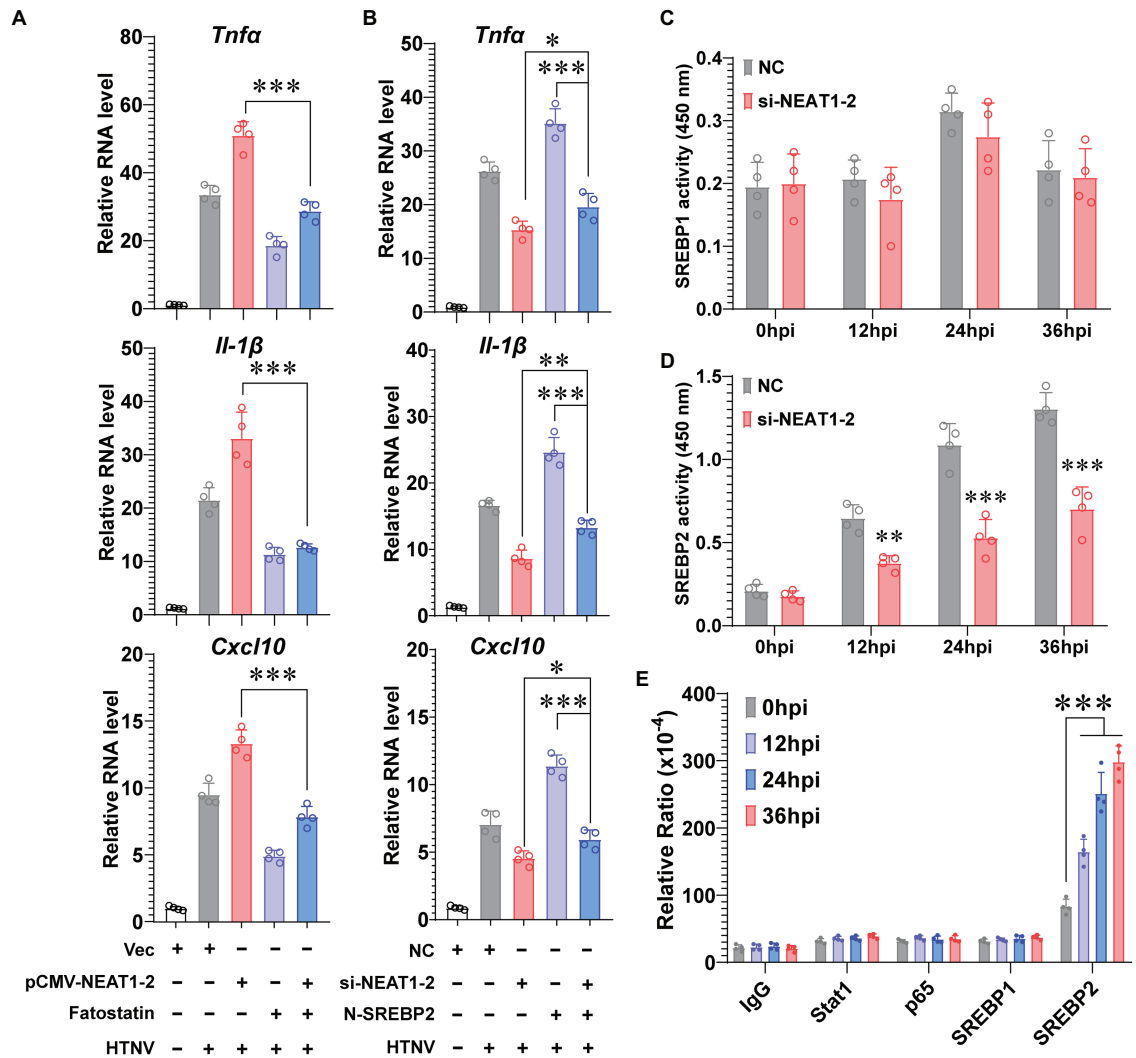
NEAT1-2 Promotes SREBP-2-Dependent Inflammation in HTNV-infected Macrophages. **(A)** qRT-PCR analysis of proinflammatory genes in hMDMs with the indicated treatments. The hMDMs were electrotransfected with pCMV-NEAT1-2 or vectors for 24 h and then infected with HTNV at an MOI of 5 with or without fatostatin (20 μM) treatment. Cells were collected for qRT-PCR at 36 hpi. **(B)** qRT-PCR analysis of proinflammatory genes in hMDMs with the indicated treatments. The hMDMs were electrotransfected with si-NEAT1-2 and/or plasmids coding N-SREBP2 and then infected with HTNV at an MOI of 5. Cells were collected for qRT-PCR at 36 hpi. **(C)** Detection of the transcriptional activity of SREBP1 in hMDMs from 0 to 36 hpi. **(D)** Detection of the transcriptional activity of SREBP2 in hMDMs from 0 to 36 hpi. **(E)** RIP assays to measure the enrichment of NEAT1-2 by different transcription factors. HEK 293T cells were transfected with plasmids expressing Stat1, p65, SREBP1 or SREBP2 and then infected with HTNV at an MOI of 5. Cells at various time points after HTNV infection were collected for RIP analysis. Data are shown as the mean ± SEM and are representative of three independent experiments. Each point represents a single sample (*n* = 4 in each group. Analysis was performed using the unpaired Student’s *t*-test **(A–D)** or one-way ANOVA **(E)**. *p < 0.05, **p < 0.01, and ***p < 0.001.

Although we have shown that NEAT1-2 could regulate SREBP2 expression (Figures 3F,G), which would further mediate host inflammatory responses against HTNV infection (Figure 4), it was uncertain whether NEAT1-2 modulated macrophage polarization mainly through SREBP2-associated signaling or through other pathways. To answer this question, the SREBP2 inhibitor fatostatin, which blocks the translocation of SREBP from the ER membrane to the Golgi (Kusnadi et al., 2019), was applied during HTNV infection. Fatostatin could largely abolish the HTNV-induced expression of inflammatory genes, such as *Tnfα*, *Il-1β*, and *Cxcl10* (Figure 5A, purple vs. gray column); notably, fatostatin almost totally reversed the strengthening effects of NEAT1-2 overexpression on cytokine production upon HTNV infection (Figure 5A, blue vs. red column), indicating that the NEAT1-2-enhanced inflammatory response in macrophages was SREBP2-dependent. Likewise, we found that exogenously expressing N-SREBP2 slightly improved HTNV-induced cytokine production in NEAT1-2-silenced hMDMs (Figure 5B, blue vs. red column), while knocking down NEAT1-2 completely reversed the proinflammatory effects of overexpressing N-SREBP2 (Figure 5B, blue vs. purple column), suggesting that the immunoregulatory role of SREBP2 in macrophages was NEAT1-2-dependent. To further assess how NEAT1-2 affects SREBP activation, the transcriptional activity of SREBP1 and SREBP2 was measured at various phases after HTNV infection. Silencing NEAT1-2 prominently suppressed the DNA-binding capacity of SREBP2 but not that of SREBP1, showing that NEAT1-2 potentiated SREBP2 activity (Figures 5C,D). Intriguingly, after exogenously expressing different transcription factors, we performed RNA immunoprecipitation (RIP) assays and found that the enrichment of NEAT1-2 by SREBP2 increased with infection progression (Figure 5E), suggesting that NEAT1-2 could interact with SREBP2. These results showed that NEAT1-2 promotes SREBP2 activation in macrophages and stimulates HTNV-induced inflammatory responses in an SREBP2-dependent manner.

### NEAT1-2 Expression Levels in Patient Monocytes Inversely Correlated With HFRS Progression

**Figure 6:**
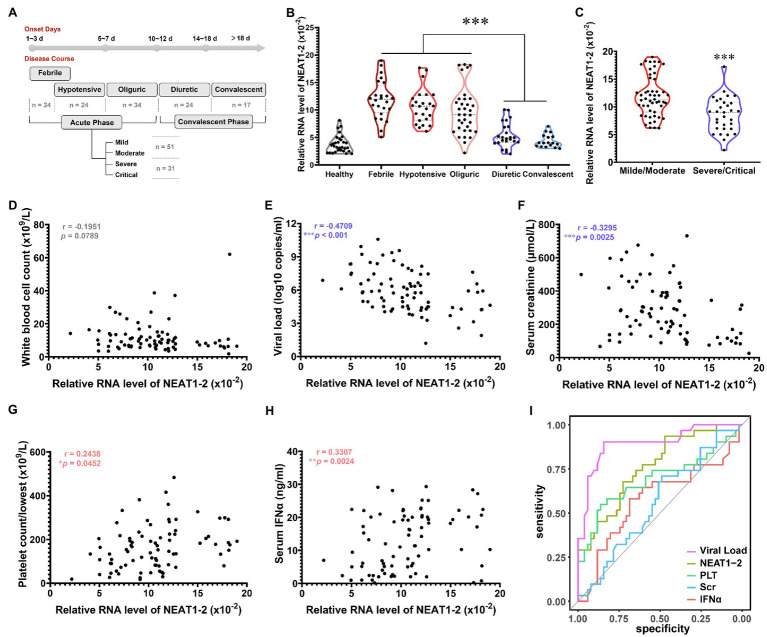
Inverse Relationship of NEAT1-2 Expression with HFRS Disease Severity. **(A)** Classification of clinical stages and disease severity for enrolled HFRS patients. Healthy control group, *n* = 30; febrile group (not combined with hypotensive shock), *n* = 24; hypotensive group (with or without fever), *n* = 24; oliguric group, *n* = 34; diuretic group, *n* = 24; convalescent group, *n* = 17. For the acute phase, mild/moderate group, *n* = 51; severe/critical group, *n* = 31. **(B)** qRT-PCR analysis of NEAT1-2 expression levels in monocytes from HFRS patients at different disease stages. **(C)** qRT-PCR analysis of NEAT1-2 expression levels in monocytes from HFRS patients at the acute phase but with different disease severities. **(D-H)** The correlation of NEAT1-2 expression level in monocytes from HFRS patients at the acute phase with white blood cell count (WBC) **(D)**, viral load **(E)**, serum creatinine concentration (Scr) (F), the lowest value of platelet count (PLT) **(G)** or serum IFNα concentration **(H)**. **(I)** Receiver operating characteristic curve (ROC) and area under the curve (AUC) analyses of the prognostic values of various clinical parameters for HFRS disease severity. Data are shown as the median, quartile, and standard deviation. Each point represents a single sample. Analysis was performed using the one-way ANOVA **(B)**, unpaired Student’s *t* test **(C)**, or Spearman’s rank correlation test **(D-H)**. *p < 0.05, **p < 0.01, and ***p < 0.001.

Having proven that NEAT1-2 could facilitate the anti-hantaviral activation of macrophages *in vitro*, we wondered whether these observations were clinically relevant. According to the clinical manifestations and related laboratory parameters of patients, the severity of HFRS can be categorized as mild, moderate, severe or critical, and the disease course can be divided into five classical stages, namely, the febrile, hypotensive shock, oliguric, diuretic, and convalescent stages, of which the former three are collectively referred to as the acute phase, while the latter two are referred to as the convalescent phase (Ma et al., 2015; Tang et al., 2019; Liu R. et al., 2021). Monocytes were acquired through negative bead screening from the peripheral blood mononuclear cells (PBMCs) of HFRS patients at different clinical stages (Figure 6A), and the NEAT1-2 expression level was calculated with the housekeeping gene *Gapdh* used for normalization. As expected, NEAT1-2 transcription was significantly elevated among patients in the acute phase of HFRS compared with healthy controls or convalescent patients (Figure 6B). Moreover, patients with mild or moderate disease tended to maintain higher NEAT1-2 expression levels than those with unfavorable prognoses (Figure 6C).

To further assess the correlation of NEAT1-2 with HFRS disease severity, Spearman’s rank correlation of NEAT1-2 with other vital clinical parameters was performed (Figures 6D–H). It has been reported that the serum viral load and creatinine (Scr) concentration, which reflects kidney injury, are negatively associated with the disease progression of HFRS, while a higher platelet count (calculated as the lowest one during the acute phase) and antiviral interferon concentration usually predict a favorable prognosis after hantavirus infection (Stoltz et al., 2007; Yi et al., 2013, 2014; Korva et al., 2019; Liu R. et al., 2021). The NEAT1-2 expression level showed no correlation with the patient white blood cell count (Figure 6D), which was also not related to disease progression, as previously reported (Yi et al., 2013). Patients with higher serum viral loads or Scr concentrations harbored lower monocyte NEAT1-2 levels (Figures 6E,F). In contrast, patients maintaining higher PLT counts or IFNα concentrations tended to possess elevated NEAT1-2 expression (Figures 6G,H). To determine the predictive values of these parameters on HFRS prognosis, the receiver operating characteristic curve (ROC) and area under the curve (AUC) were analyzed. The viral load showed the most meaningful diagnostic value (AUC = 0.894), followed by PLT count (AUC = 0.691), IFNα concentration (AUC = 0.578), and Scr concentration (AUC = 0.58). Of note, the NEAT1-2 level showed a relatively better diagnostic value (AUC = 0.761; Figure 6I). These data revealed that the NEAT1-2 expression level of patient monocytes correlated with disease parameters and could be used for the evaluation of immune function and antiviral status in HFRS patients.

## Discussion

**Figure 7:**
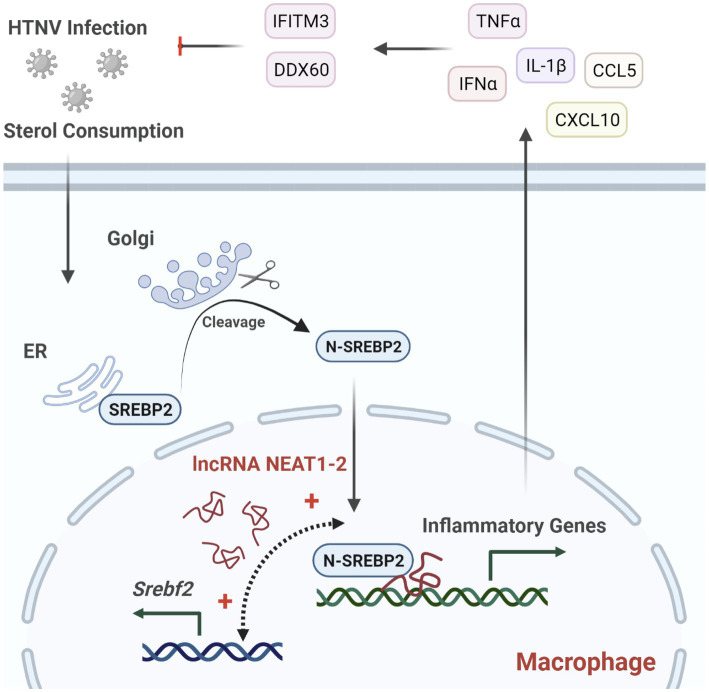
Regulation of NEAT1-2 in SREBP2-mediated Anti-hantaviral Macrophage Responses. HTNV infection consumes cellular sterols and activates the SREBP2 pathway in macrophages, during which lncRNA NEAT1-2 potentiates SREBP2 activity by facilitating *Srebf1* expression and initiating SREBP2-mediated inflammation. M1-type macrophages further stimulate host antihantaviral responses by secreting multiple cytokines, including IFNα, TNFα, and IL-1β. These cytokines promote the expression of antiviral molecules such as IFITM3 and DDX60, thus restricting HTNV replication and spread.

The innate immune system constitutes the first line of defense against pathogen infection, of which monocytes and macrophages are reported to be subverted by viruses for their spread and disease progression (Baasch et al., 2021; Huang, 2021; Rojas et al., 2021). Investigating how host antiviral responses are launched and regulated might provide potential therapeutic targets for limiting viral infection and propagation. In this study, we identified the lncRNA NEAT1-2 in monocytes and macrophages as an earlier responder to HTNV infection. In HTNV-infected macrophages, NEAT1-2 promotes the expression of SREBP2, a key transcription factor for cholesterol synthesis, and then binds to SREBP2 and potentiates its activity, thus controlling the metabolic phenotype changes and initiating robust inflammatory responses to limit hantaviral propagation (Figure 7). We demonstrate that the host NEAT1-2-SREBP2-cytokine gene axis acts as a novel mechanism to mediate inflammatory macrophage polarization after HTNV infection and show that the NEAT1-2 expression level might be a relatively easily assessed biomarker that is inversely correlated with HFRS severity.

Recently, an increasing number of studies have proven that lncRNAs, which are transcripts with a length of more than 200 nucleotides and without the coding protein ability, expressed by a viral host can regulate multiple immunological and metabolic pathways upon infection by acting as guides, decoys, or scaffolds (Ren et al., 2021; Sajjad et al., 2021; Yang Q. et al., 2021). Type I IFN and its downstream signaling pathway components are modulated in two ways by host lncRNAs. On the one hand, the lncRNA-ISIR is induced by type I IFN and exerts positive feedback by binding interferon regulatory factor 3 (IRF3), which is a vital transcription factor for IFN production, and enhancing its phosphorylation and activation (Xu J. et al., 2021). On the other hand, the lncRNA MALAT1 interacts with the transactive response DNA-binding protein (TDP43) and blocks its cleavage into TDP35, thus suppressing IRF3 activity by promoting its degradation (Liu et al., 2020). The lncRNA NRAV can also inhibit the expression of several critical interferon-stimulated genes (including Mx1 and IFITM3) by affecting their histone modification or facilitate Rab5c-mediated vesicle transportation by detaching microRNA miR-509-3p from Rab5c, thus assisting influenza virus or respiratory syncytial virus replication, respectively (Ouyang et al., 2014; Li et al., 2020). Alternatively, host lncRNAs might exert IFN-independent effects on viral replication. The lncRNA ACOD1, which is activated by infection with multiple viruses but not by type I IFN stimulation, can bind to and enhance the catalytic activity of the metabolic enzyme glutamic-oxaloacetic transaminase (GOT2), the activity of which is indispensable for viral replication (Wang et al., 2017). The lncRNA NORAD might trigger cytokine storms during SARS-CoV-2 infection by promoting the production of IL-6, IL-10, CSF3, TNFα, and CXCL10 (Morenikeji et al., 2020; Yang Q. et al., 2021). NORAD also interacts with miR-373 and Wee1 to favor cell growth of hepatocytes and facilitate chronic infection of hepatitis C virus (Sur et al., 2018). The lncRNA GM was observed to bind to glutathione S-transferase M1 (GSTM1) and block the GSTM1-mediated S-glutathionylation of TANK-binding kinase 1 (TBK1), thus facilitating TBK1 activation and strengthening the transcription of multiple proinflammatory cytokines (Zhou et al., 2020). The lncRNA GAS5, which accumulates in infectious diseases, could function as a potent repressor of the glucocorticoid receptor (GR) and occlude the anti-inflammatory effects of endogenous glucocorticoids (Mayama et al., 2016). Although most lncRNAs have been shown to magnify host antiviral immune responses, previous studies have also indicated that several lncRNAs act as important repressors of inflammation to prevent cytokine storms and ameliorate tissue immunopathogenesis. The lncRNA TUG1 was shown to relieve host inflammatory responses *via* the miR-494/PDK4 axis and mitigate LPS-induced acute lung injury (Yang L. et al., 2021). Here, we discovered through RNA-seq that the transcription of many lncRNAs, such as NEAT1, MALAT1, GAS5 and TUG1, was induced in HTNV-infected macrophages, among which NEAT1 showed an earlier response with relatively higher endogenous expression (Figure 1).

NEAT1-2, the longer transcript of NEAT1, is mainly located in the nucleus and has been reported to facilitate IL-8 and CCL5 production by sequestering SFPQ in paraspeckles after infection with various viruses (Imamura et al., 2014). We previously showed that NEAT1 was activated by HTNV through the RIG-I-IRF7 pathway, which in turn positively contributed to RIG-I- and DDX60-mediated IFN generation *via* a similar mechanism (Ma H. et al., 2017). In contrast, we discovered that NEAT1-2 potentiated M1 polarization by directly binding SREBP2 and promoting SREBP2-mediated cytokine production (Figure 5), which indicated a paraspeckle-independent regulatory function of NEAT1-2. For the detailed mechanism, it is possible that NEAT1-2 selectively recruited SREBP2 to the inflammatory genes in the nucleus. However, it is worth noting that NEAT1-2 also translocates into the cytoplasm and facilitates inflammasome formation in macrophages (Zhang et al., 2019). We could not exclude the possibility that NEAT1-2 targeted SREBP2 in the cytoplasm and assisted its activation. Hence, further investigation is required to clarify the subcellular location of their interaction (whether in the nucleus or cytoplasm) and their specific binding motif. On the other hand, considering that NEAT1-1, the shorter isoform of NEAT1, was shown to augment cellular glycolysis (Park et al., 2021) and that the carbohydrate mechanism pathway was enriched during HTNV infection (Figure 1B, upper), it remains to be discussed whether NEAT1-1 affects HTNV-stimulated macrophage polarization by regulating glycolysis. Moreover, it was reported that the altered expression of NEAT1 in PBMCs was negatively correlated with dengue severity and could discriminate mild dengue infection (DI) from severe dengue shock syndrome (DS; Pandey et al., 2017). We found that the NEAT1-2 expression level in patient monocytes also exhibited an inverse correlation with HFRS progression (Figure 6). Considering that HFRS patients might receive dialysis treatment or continuous renal replacement therapy (Liu et al., 2019), which would affect the results of serum parameter measurements, NEAT1-2 in monocytes could be a better biomarker than serum viral load or PLT count.

Under physiological conditions, the predominant function of SREBP2 in macrophages is to maintain the basic enzyme gene expression for cholesterol synthesis that is elaborately regulated by cellular sterol metabolites. SREBP-mediated lipid metabolism is highly involved in the life cycle of hantavirus, as sterol synthesis and related membraned fusion processes are indispensable for hantaviral entry and progeny virus release (Petersen et al., 2014; Kleinfelter et al., 2015). Nevertheless, little is known about how SREBP regulates host anti-hantaviral immune responses. The crosstalk between metabolism and immune activation is mediated by metabolites, which could control immune cell expansion and/or effector function (O'Neill et al., 2016). According to this predominant model of the immunometabolism field, it is well acknowledged that SREBPs influence immune cell activation by producing cholesterol or the molecules generated from such processes (such as cholesterol pathway intermediates or cholesterol-derived oxysterols; Dang et al., 2017; Bekkering et al., 2018). Another perspective is that the sterols downstream of SREBPs could manipulate immune responses directly, by interacting with certain cellular receptors or signaling adaptors, or indirectly, by altering the membrane composition and hence affecting their subcellular localization (Fessler, 2016). Recent research revealed that SREBP2 was predominantly activated in TNFα-stimulated macrophages and enhanced sterol-independent M1 polarization by directly binding to and triggering the transcriptional activation of inflammatory and IFN response genes (Kusnadi et al., 2019). Our study partially clarified the mechanism by which SREBP2 changed its genomic binding profile to include inflammatory and IFN response genes, the process of which might be mediated by lncRNA NEAT1-2. We also discovered that various inflammatory cytokines and chemokines were manipulated by SREBP2 in addition to TNFα, IL-1β, CCL5, CXCL10 and CXCL11, as a previous study indicated (Kusnadi et al., 2019). It is also clear that although HTNVs might hijack cholesterol metabolism at the early infection stage for their entry and replication, the integrated effects of the stimulated SREBP2 pathway in macrophages are antihantavirus but not proinfection, as we showed in Figure 4.

There also exist some limitations in this study. We found that the viral RNA alteration seemed to be modest at the early infection stage (12 hpi) after silencing NEAT1-2, while the difference would be augmented at the late stage (36 hpi; Figure 2D). Adding more checking time points, such as from 48 to 72 hpi, might be better to evaluate the influence of NEAT1-2 on hantaviral replication. Another limitation is that we did not measure how the non-pathogenic hantavirus, such as the Tula or Prospect Hill virus, affected NEAT1-2 expression. Although previous RNA-seq data showed that the Tula virus infection could not induce NEAT1 expression in A549 cells (GEO accession: GSE73410), it remains to be arbitrary to conclude that only pathogenic HTNV triggers NEAT1-2-SREBP2-cytokine gene expression. It would be of great importance to compare the responses between the pathogenic and non-pathogenic hantaviruses. Additionally, it could not be ignored that though the Sulforaphane might enhance inflammatory responses by inducing NEAT1-2 expression, this isothiocyanate could also restrain the activation of NF-κB pathway and inhibit the production of reactive oxygen species, thus partially restricting the innate antiviral effects. That is why the antihantaviral effects of SFN were not quite prominent from the biological aspects even though the statistical analysis showed significance (Supplementary Figures S1B,C). Considering the complex biological effects, more experiments, especially the antiviral effects of Sulforaphane *in vivo*, should be evaluated in the future.

In conclusion, we found in this study that lncRNA NEAT1-2 regulated HTNV-induced M1 polarization, constraining viral propagation and spread between cells. NEAT1-2 not only promoted SREB2 expression and cholesterol synthesis at the early infection phase but also interacted with SREBP2 and strengthened SREBP2-mediated inflammatory responses. Moreover, NEAT1-2 expression in monocytes was shown to be negatively correlated with HFRS progression. These results revealed a novel biomarker for the evaluation of host immune functions against HTNV infection and suggested a potential therapeutic target for the clinical treatment of HFRS.

## Data Availability Statement

The datasets presented in this study can be found in online repositories. The names of the repository/repositories and accession number(s) can be found at: ArrayExpress—E-MTAB-11353.

## Ethics Statement

The studies involving human participants were reviewed and approved by the Ethical Committee of the Fourth Military Medical University and the Institutional Review Board of Tangdu Hospital (TDLL-2016323). The patients/participants provided their written informed consent to participate in this study.

## Author Contributions

HM, FZ, and LS conceptualized the study and designed the experiments. YY, ML, and YM conducted the experiments. WY and YS helped with the literature research and analyzed the experimental data. XZ, HL, and LC collected the patient samples and analyzed the clinical data. LZ and HZ prepared the manuscript and performed data analysis. XZ and YL assisted with the manuscript revision and final approval for submission. All authors contributed to the article and approved the submitted version.

## Funding

The present study was supported by grants from the National Natural Science Foundation of China (82172272 and 31970148) and the Discipline Devoting Project of Xijing Hospital (XJZT21CM09).

## Conflict of Interest

The authors declare that the research was conducted in the absence of any commercial or financial relationships that could be construed as a potential conflict of interest.

## Publisher’s Note

All claims expressed in this article are solely those of the authors and do not necessarily represent those of their affiliated organizations, or those of the publisher, the editors and the reviewers. Any product that may be evaluated in this article, or claim that may be made by its manufacturer, is not guaranteed or endorsed by the publisher.

## Footnotes

^1^ www.omicshare.com
