# Supplementary figures and images for: LncRNA NEAT1 Potentiates SREBP2 Activity to Promote Inflammatory Macrophage Activation and Limit Hantaan Virus Propagation
Source: Front Microbiol. 2022 Apr 13;13:849020. doi: 10.3389/fmicb.2022.849020 (PMC9044491; doi:10.3389/fmicb.2022.849020)

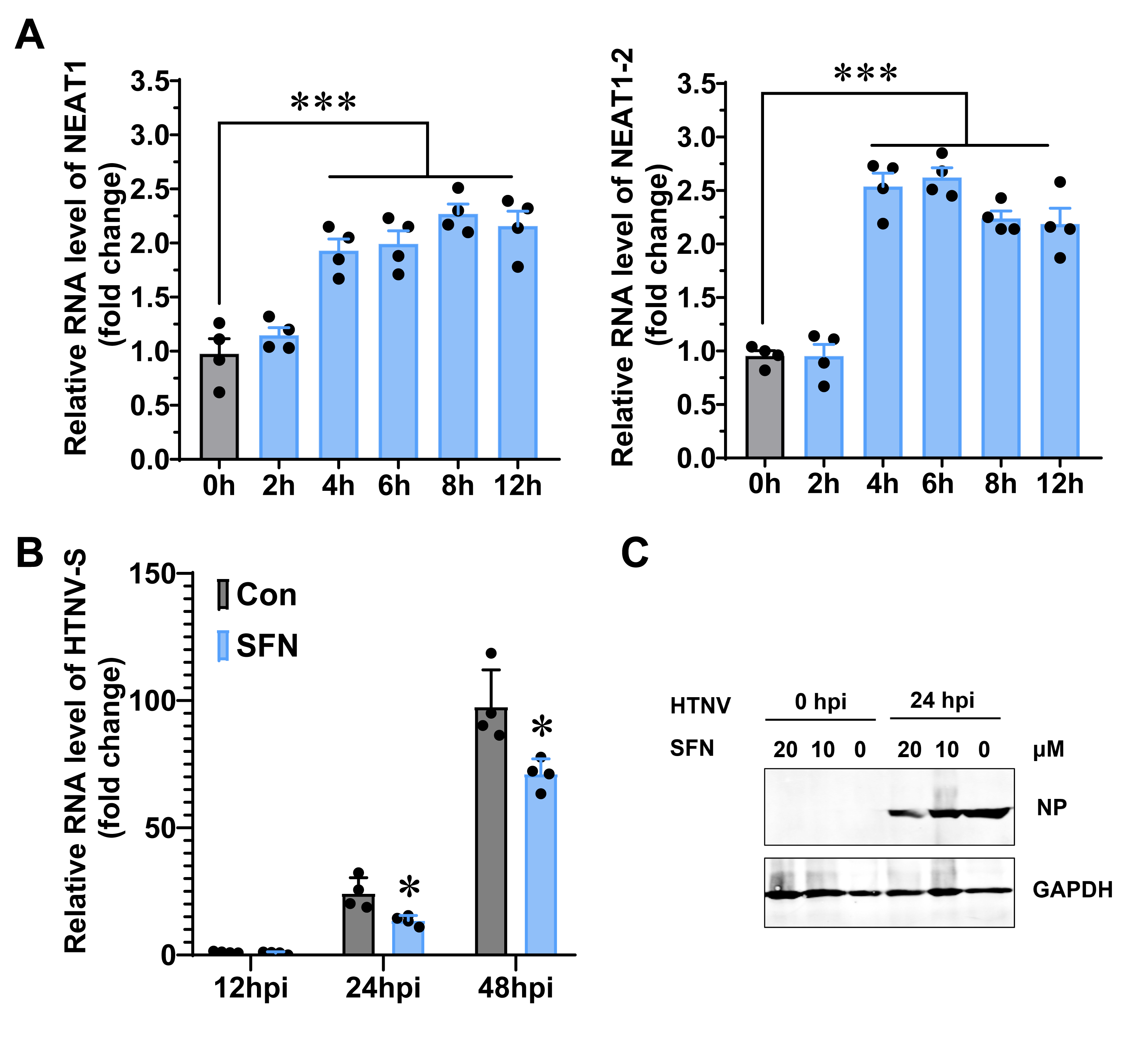

Supplement: Supplementary file 1 [file Image_1.tif]
